# A novel inducible CRISPRi tool, CRISPRi‐Cre, to study neuron‐specific phenotypes in iPSC‐derived neuron models of Alzheimer's Disease

**DOI:** 10.1002/alz70855_097245

**Published:** 2025-12-23

**Authors:** Daniel M Ramos, Matthew P Nelson, Liz Calzada, Sanjana Krishna, Samuel S Neuman, Cory Weller, Nicholas Johnson, Mike A Nalls, Erika Lara, Andy Qi, Marianita Santiana, Caroline Pantazis, William C Skarnes, Andrew Singleton, Mark R Cookson, Michael Ward

**Affiliations:** ^1^ Center for Alzheimer's and Related Dementias, National Institute on Aging, National Institutes of Health, Bethesda, MD, USA; ^2^ Data Tecnica, Washington DC, DC, USA; ^3^ Data Tecnica, Bethesda, MD, USA; ^4^ National Institute on Aging, Bethesda, MD, USA; ^5^ Data Tecnica International, Washington, DC, USA; ^6^ Center for Alzheimer's and Related Dementias, National Institute on Aging and National Institute of Neurological Disorders and Stroke, National Institutes of Health, Bethesda, MD, USA; ^7^ The Jackson Laboratory, Farmington, CT, USA; ^8^ Cell Biology and Gene Expression Section, Laboratory of Neurogenetics, National Institute on Aging, National Institutes of Health, Bethesda, MD, USA

## Abstract

**Background:**

The iPSC Neurodegenerative Disease Initiative (iNDI) is the largest‐ever induced pluripotent stem cell (iPSC) genome engineering project, modeling over 100 ADRD mutations in high‐quality isogenic human iPSCs. iNDI leverages unbiased CRISPRi screens as a powerful tool to identify fundamental mechanisms and modifiers of disease. However, current CRISPRi molecular tools are poorly optimized for use in iPSC‐derived neurons (iNeurons). Here we develop a Cre‐lox inducible CRISPRi system (CRISPRi‐Cre), enabling gene knockdown upon Cre delivery to postmitotic iNeurons, and identification of neuron‐specific, disease‐relevant modifiers.

**Method:**

We modified a plasmid carrying a potent Zim3‐dCas9 transcriptional repressor to include a strong floxed STOP cassette upstream of the Zim3 start codon. We leveraged HaloTag‐TDP43 and HaloTag‐FUS iSPCs from the iNDI project paired with flow cytometry to validate leakiness and responsiveness to Cre in iPSCs and iNeurons treated with sgRNAs. We then performed a genome‐wide CRISPRi survival screen in iNeurons to demonstrate broad functionality of this inducible CRISPRi system with over 20,000 sgRNAs. Finally, we use CRISPRi‐Cre to identify neuron‐specific regulators of neuronal activity in iNeurons.

**Result:**

We demonstrate that in the absence of Cre, dCas9 is inactive. Delivery of lentivirus‐Cre to iNeurons activates dCas9, resulting in potent gene knockdown. In genome‐wide CRISPRi screens, we show that CRISPRi‐Cre identifies many of the same hits observed in screens using constitutive‐active dCas9, and importantly uncovers novel neuron‐specific hits not identified in previous CRISPRi screens.

**Conclusion:**

Here, we developed a robust Cre‐inducible CRISPRi system that enables post‐mitotic gene knockdown in iPSC‐derived neurons. Our CRISPRi screens identify neuron‐specific hits, demonstrating the utility of our tool to help uncover disease‐relevant mechanisms, modifiers, and potential therapeutic targets in relevant cell types.

